# Cerebral glucose changes after chemotherapy and their relation to long-term cognitive complaints and fatigue

**DOI:** 10.3389/fonc.2022.1021615

**Published:** 2022-10-13

**Authors:** Gwen Schroyen, Georg Schramm, Donatienne Van Weehaeghe, Nicolas Leenaerts, Thomas Vande Casteele, Jeroen Blommaert, Michel Koole, Ann Smeets, Koen Van Laere, Stefan Sunaert, Sabine Deprez

**Affiliations:** ^1^ Leuven Brain Institute, KU Leuven, Leuven, Belgium; ^2^ Leuven Cancer Institute, KU Leuven, Leuven, Belgium; ^3^ Department of Imaging and Pathology, Translational MRI, KU Leuven, Leuven, Belgium; ^4^ Department of Imaging and Pathology, Nuclear Medicine and Molecular Imaging, KU Leuven, Leuven, Belgium; ^5^ Division of Nuclear Medicine, University Hospitals Leuven, Leuven, Belgium; ^6^ Department of Neurosciences, Mind-Body Research, KU Leuven, Leuven, Belgium; ^7^ University Psychiatric Centre, KU Leuven, Leuven, Belgium; ^8^ Department of Psychiatry, University Hospitals Leuven, Leuven, Belgium; ^9^ Department of Neurosciences, Neuropsychiatry, KU Leuven, Leuven, Belgium; ^10^ Department of Oncology, Gynaecological Oncology, KU Leuven, Leuven, Belgium; ^11^ Department of Oncology, Surgical Oncology, KU Leuven, Leuven, Belgium; ^12^ Surgical Oncology, University Hospitals Leuven, Leuven, Belgium; ^13^ Radiology, University Hospitals Leuven, Leuven, Belgium

**Keywords:** neuroimaging, breast cancer, chemotherapy, metabolism, FDG-PET, cognitive complaints, fatigue

## Abstract

**Purpose:**

To investigate the short-term cerebral metabolic effects of intravenous chemotherapy and their association with long-term fatigue/cognitive complaints.

**Experimental design:**

Using [^18^F]-FDG-PET/CT whole-body scans, we retrospectively quantified relative cerebral glucose metabolism before and after neoadjuvant chemotherapy in a cohort of patients treated for non-metastatic breast cancer (2009-2019). Self-report of cognitive complaints and fatigue were prospectively assessed 7 ± 3 years after therapy. Metabolic changes were estimated with i) robust mixed-effects modelling in regions-of-interest (frontal, parietal, temporal, occipital, and insular cortex) and ii) general-linear modelling of whole-brain voxel-wise outcomes. iii) The association between metabolic changes and self-reported outcomes was evaluated using linear regression-analysis.

**Results:**

Of the 667 screened patients, 263 underwent PET/CT before and after chemotherapy and 183 (48 ± 9 years) met the inclusion criteria. After chemotherapy, decreased frontal and increased parietal and insular metabolism were observed (|ß|>0.273, *p_FDR_* <0.008). Separately, additional increased occipital metabolism after epiribucin+ cyclophosphamide (EC) and temporal metabolism after EC+ fluorouracil chemotherapy were observed (ß>0.244, *p_FDR_* ≤0.048). Voxel-based analysis (*p_cluster-FWE_* <0.001) showed decreased metabolism in the paracingulate gyrus (-3.2 ± 3.9%) and putamen (3.1 ± 4.1%) and increased metabolism in the lateral cortex (L=2.9 ± 3.1%) and pericentral gyri (3.0 ± 4.4%). Except for the central sulcus, the same regions showed changes in EC, but not in FEC patients. Of the 97 self-reported responders, 23% and 27% experienced extreme fatigue and long-term cognitive complaints, respectively, which were not associated with metabolic changes.

**Conclusion:**

Both hyper- and hypometabolism were observed after chemotherapy for breast cancer. Combined with earlier findings, this study could support inflammatory mechanisms resulting in relative hypermetabolism, mainly in the parietal/occipital cortices. As early metabolic changes did not precede long-term complaints, further research is necessary to identify vulnerable patients.

## Introduction

Owing to the growing survival rates after cancer treatment, there has been increased interest in the risk factors and mechanisms for post-treatment side effects. For instance, cancer-related cognitive impairment is observed in up to 75% of patients with breast cancer during treatment (1) and persisting years after chemotherapy in a subset (2, 3). Additionally, while most women report fatigue throughout treatment, one subgroup is at risk of severe or persistent fatigue (4). These subtle side effects can have a negative impact on quality of life and work ability, resulting in indirect societal costs (5).

Several mechanisms have been proposed to moderate neurocognitive symptoms. The toxic effects of chemotherapy on organs, DNA damage, and the crossing of cytostatic agents through the blood-brain barrier (potentially initiating brain damage) are all proposed direct mechanisms (6, 7). Given the limited availability of chemotherapeutics in the brain and the chronic nature of these side effects, indirect mechanisms have been suggested (6). These include psychological/functional moderators (e.g., anxiety and fatigue) and cytokine-induced neuroinflammation (6). Our group recently demonstrated that patterns of microglial activity, indicative of neuroinflammation, differ between patients who have undergone chemotherapy and controls (8). Moreover, higher frontal glial hyperactivity was associated with worse cognitive performance, suggesting neuroinflammation as an underlying mechanism for the complaints observed in this population (8).

Currently, there are no standardized diagnostic tests available to identify patients at risk for cognitive and psychological complaints. As recruitment for additional study-specific assessments can be challenging, with patients coping with cancer diagnosis and upcoming treatment, available clinical data could provide initially useful information. In an oncologic setting, *whole-body* [(18)F]-fluorodeoxyglucose (FDG) positron-emission tomography (PET) combined with computed tomography (CT) imaging is used for staging and therapeutic tumoral response-evaluation (9). Cerebral-FDG, a glucose analog, has been viewed as a proxy for neuronal activity (10–12) but also inflammation (13–15). Therefore, clinical FDG-PET before and after chemotherapy can be valuable in determining alterations in chemotherapy-induced brain metabolism.

Dedicated FDG-PET neuroimaging studies have served as a powerful tool for identifying resting brain activity associated with a variety of conditions affecting cognition (16). In patients with breast cancer, cross-sectional studies found that one (17) and five to ten years (18) post-chemotherapy brain metabolism correlated with memory outcome, while this was not the case 16 years post-treatment for other cognitive domains (19). To date, no longitudinal brain FDG-PET studies have been performed in patients with breast cancer undergoing chemotherapy. However, when using available sequential whole-body FDG-PET/CT in a routine setting, decreased glucose consumption was observed in non-small-cell lung cancer, widespread throughout the brain post-chemotherapy (20). This indicates the feasibility of such an approach, despite short scan times (typically 2-3 minutes), for the cerebral single-bed acquisition (21).

It remains unclear whether long-term cognitive/psychological complaints after chemotherapy are preceded by cerebral metabolic changes. Therefore, this study aimed to investigate changes in cerebral glucose metabolism after neoadjuvant chemotherapy for breast cancer and their association with complaints years later. We retrospectively analyzed the gray matter (GM) regions of interest using clinical whole-body FDG-PET/CT imaging and prospectively collected self-reports of complaints.

## Methods

### Participants

**Figure 1:**
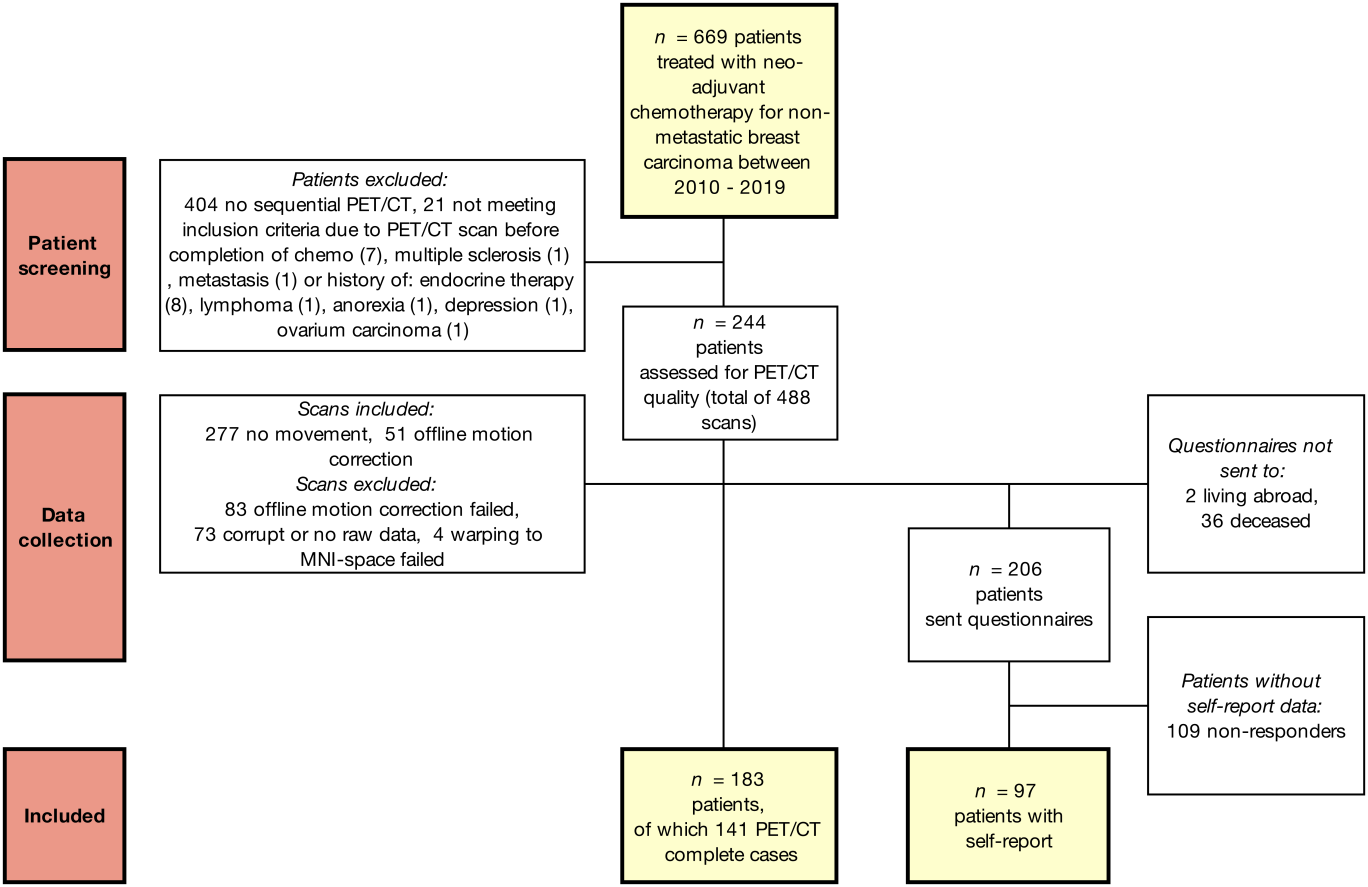
Flow chart of included patients and data. Robust mixed-effects modelling ensured incomplete cases could also be included in the statistical models.

A database was compiled from the electronic medical records of patients aged <65 years diagnosed with non-metastatic breast carcinoma between January 2009 and October 2019 at the University Hospitals Leuven. Women who underwent FDG-PET/CT imaging as part of cancer staging before therapy (T_0_) and follow-up after receiving neoadjuvant chemotherapy (T_1_) were included. The standard clinical whole-body PET/CT protocol includes imaging from the thighs to the skull, including the brain. This creates a useful database for longitudinal evaluation of brain metabolism changes. Women with a history of other cancers (treatment) or a clinical diagnosis of a psychiatric/neurological condition/injury/intellectual disability were excluded. Eligible survivors were sent an informative letter for this retrospective study according to the GDPR guidelines, and additional questionnaires were mailed (**Figure 1**). The responders provided written informed consent for the use of the questionnaires. This study was approved by the local ethics committee and was conducted in accordance with the Declaration of Helsinki.

### Clinical imaging and processing

The participants underwent whole-body FDG-PET/CT scanning using Siemens Biograph 16 (10%), Siemens Biograph 40 (81%; Siemens Healthcare), or GE Discovery MI (8%; GE Healthcare), starting 47-153 minutes (mean = 80, 95% confidence interval (CI) = 78 - 82) after intravenous [^18^F]FDG injection (mean 298, CI = 294 - 306 MBq). All subjects were instructed to fast for at least six hours before imaging, and blood glucose concentration was measured before tracer injection (mean = 96, CI = 94 - 96) mg/dL). All subjects rested quietly during the FDG uptake period with the instruction to keep their eyes open in a dark room, according to the standard clinical protocol. PET scans of either 130, 160, or 180s per bed position were acquired in the three-dimensional mode, and examinations included low-dose CT acquisition (120 kV, 11mAs) for attenuation correction. [^18^F]FDG whole-body images were reconstructed using iterative ordered subset expectation maximization (OSEM) for the Biograph 40 (3 iterations, 24 subsets, 5 mm FWHM Gaussian post filter) and Biograph 16 (5 iterations, 8 subsets, 6 mm FWHM Gaussian post filter) systems or regularized Q. Clear (penalized likelihood) reconstruction for the Discovery MI (regularization parameter beta=600). All [^18^F]FDG-PET scans were first visually evaluated for a mismatch between PET and CT using MIMVista (MIM-Software Inc.) by a nuclear medicine fellow (DVW). For cases showing a motion-induced mismatch between the PET brain position and CT, a new reconstruction was performed with the head part of the CT first rigidly aligned to a PET reconstruction of the brain position without attenuation correction (by optimizing the mutual information between the CT and PET reconstruction without attenuation correction), prior to the final OSEM reconstruction with attenuation correction (3 iterations, 24 subsets, 5 mm FWHM Gaussian post filter).

For the quantification of glucose metabolism, region-of-interest (ROI) and voxel-based analyses were performed with relative glucose metabolism as the main parameter of interest by scaling each image to the average cortical GM uptake. A GM mask was created with a tissue probability map [SPM12 (22)] thresholded at 0.5. FDG uptake images were spatially registered to an in-house available mean FDG image (2 mm isotropic) in the MNI space (rigid + affine + deformable syn transform with mutual information using ANTs [23)]. Composite volumes of interest containing five bilateral regions, based on the Hammers N30R83 atlas, were subsequently used as masks to calculate the mean values in the ROIs: frontal, temporal, occipital, parietal, and insular cortices (calculations with MRtrix3 [24)], corresponding to earlier cortical ROIs investigated for neuroinflammation (8). Voxel-based analyses were performed for complete cases, if any differences were observed. FDG images were smoothed with an isotropic Gaussian kernel of 10 mm FWHM (with MRtrix3).

### Clinical parameters and self-report

Clinical parameters (**Table 1**) were extracted from the electronic patient files. Survivors were sent the Fatigue Assessment Scale (FAS; score ≥22 indicates substantial fatigue and ≥35 extreme fatigue) (25) and the Cognitive Failure Questionnaire [CFQ; total score ≥45 indicates complaints and ≥55 indicates severe complaints (26, 27)].

**Table 1:** Demographics and characteristics of the study population.

Characteristic	Entire sample (*n* = 183)		Self-report subsample (*n* = 97)	
	Mean or *n*	95% CI (range) or (%)	Mean or *n*	95% CI (range) or (%)
Age (years)	48	47 - 49 (range 27 – 65)	49	47 – 51 (range 27 – 65)
Time T0 to start chemotherapy (days)	13	12 – 14 (range 0 – 37)	12	11 – 13 (range 2 – 27)
Time end chemotherapy to T1 scan (days)	10	9 – 11 (range 0 - 26)	10	9 – 12 (range 0 – 26)
Chemotherapy duration (days)	126	124 - 129 (range 65 – 245)	127	121 – 130 (range 84 – 182)
Time since end of chemotherapy (years)	7	6.1 – 6.7 (range 2 – 12)	7	5.9 – 7.1 (range 2 – 12)
Body-mass index (kg/m^2^)	25	24 – 26 (range 16 – 43)	26	23 – 26 (range 18 – 43)
*Menopausal status at diagnosis:				
Pre	100	(59)	48	(57)
Peri	11	(7)	7	(8)
Post	58	(34)	29	(34)
Breast cancer stage:				
0-1	8	(4)	3	(0)
2	75	(41)	42	(40)
3	100	(54)	51	(53)
Cancer treatment:				
EC + T chemotherapy	100	(54)	53	(55)
FEC + T chemotherapy	83	(46)	44	(45)
Neoadjuvant HER2 blocker	59	(32)	36	(37)
Adjuvant HER2 blocker	45	(25)	26	(27)
Adjuvant radiotherapy	171	(93)	90	(94)
Adjuvant endocrine therapy	100	(55)	54	(57)
of which tamoxifen†	47	(47)	23	(43)
of which aromatase inhibitor†	89	(89)	48	(89)
Self-report:				
Fatigue assessment scale (total)	–		29	28 – 31 (range 13 – 48)
Fatigue, score ≥ 22	–		78	(81)
Extreme fatigue, score ≥ 35	–		22	(23)
Cognitive failure questionnaire (total)	–		41	37 – 44 (range 4 – 79)
Cognitive complaints, score ≥ 45	–		37	(39)
Extreme cognitive complaints, score ≥ 55	–		26	(24)

*Menopausal status was known in 92% of the entire sample and 87% of the self-report subsample. Adjuvant therapy was administered after a T1 PET scan. † Aromatase inhibitors include anastrazole, exemestane, and letrozole. The combination of an aromatase inhibitor and tamoxifen is also possible. CI, confidence interval; EC, 4 rounds of epirubicin 90 mg/m^2^ + cyclophosphamide 600 mg/m^2^; FEC = 3-6 rounds of 500 mg/m^2^ 5-fluorouracil and 4 rounds of epirubicin 100 mg/m^2^ + cyclophosphamide 500 mg/m^2^; T = 1-12 rounds of paclitaxel 80 mg/m^2^ .

### Statistical analysis

Clinical and ROI statistical analyses were performed using R v4.1.2. The normality of the data was assessed and logarithmically transformed when it was non-Gaussian. The Wilcoxon signed-rank test was used to assess the differences in the injected tracer dose and time to the start of scans after injection between time points.

To investigate the effects of time on relative glucose metabolism, two analyses were carried out to i) evaluate macroscopic changes in the full dataset and ii) add more regional specificity to these changes for complete cases. First, robust linear mixed-effects models were estimated (DAStau method) per ROI (robustlmm-package, version 2.4-5). With robust linear mixed effects modelling, we were able to account for repeated measures and missingness in the data, still including single-timepoint data (mixed effects) (28) as well as provide estimates where outliers or other contamination have little influence, especially relevant in large datasets (robust) (29). These models included relative ROI glucose metabolism as a dependent variable, time point of scan, chemotherapy regime as fixed effects, and an interaction between time point and chemotherapy regime corrected for age, BMI, scanner, years since chemotherapy, and days between scans. A random intercept per subject was fitted to account for repeated measures. Fixed effects were removed when they were not significantly aiding to any of the models, and the models were re-run. Continuous variables were standardized for easier model interpretation, estimates can therefore be interpreted as effect sizes. Statistical significance was determined with false discovery rate correction (Benjamini-Hochberg) to compare the five regions of interest at *p_FDR_* <.05. Second, in SPM12, a generalized linear model was used to explore time effects (flexible factorial), including subject, time of scan, chemotherapy regimen, and scanner as factors, and age as covariate. To add regional specificity to the ROI results, statistical significance was inferred with a stringent height threshold of familywise-error correction for multiple comparisons of *p*_FWE_<.05 and cluster-level *p*_FWE_<.001. Thirdly, for the regions showing voxel-wise time differences (both treatment groups combined), linear regression models were computed to evaluate the association of cognitive complaints or fatigue based on percent mean relative glucose metabolism changes [(PET T_1_–PET T_0_)/PET T_0_] in the cluster or baseline (PET T_0_) metabolism. The models included chemotherapy regimen, endocrine therapy (yes/no), radiotherapy (yes/no) and HER2 therapy (yes/no) as fixed factors and were corrected for age and years since chemotherapy. Fixed effects were removed when they were not significantly aiding to any of the linear models, and the models were re-run. Continuous variables were also standardized for easier model interpretation and statistical significance was inferred with *p_FDR_* <.05 for comparing several voxel-clusters (here: four).

## Results

### Participants

Of the 669 patients who received neoadjuvant chemotherapy between 2010 and 2019, 265 underwent two clinical whole-body PET/CT scans (**Figure 1**). Of these, 21 were excluded for not meeting the inclusion criteria, 36 (14%) were deceased and two lived abroad. The remaining 206 women were sent questionnaires, and 97 (47%) responded. Data inspection revealed severe movement between the CT and PET scans in 134 patients. Of these, 51 scans were successfully motion corrected using automated processing. Warping to the MNI space failed in four scans. The final sample included 183 chemotherapy-treated patients for whom PET/CT scans with no visual movement were available, with 141 (77%) patients for whom two scans were available. All 183 patients underwent neoadjuvant chemotherapy with epirubicin+cyclophosphamide+taxanes (54%) or additional 5-fluorouracil (46%). As can be seen from the demographic and clinical characteristics (**Table 1**), the self-report subsample showed good correspondence with the entire sample.

### Systemic chemotherapy induces differential metabolic patterns

**Figure 2:**
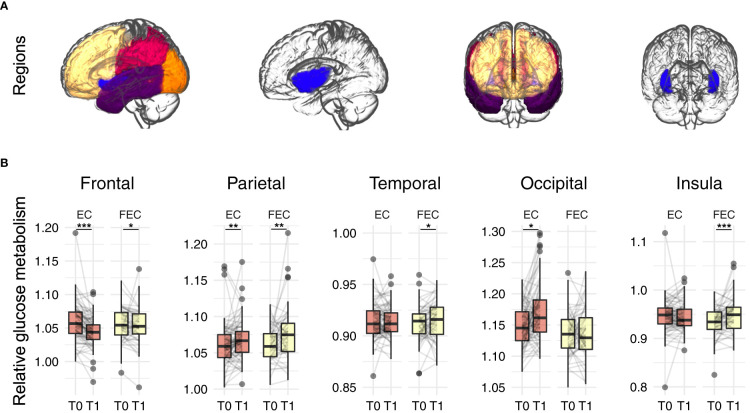
Regions of interest analysis of relative glucose metabolism before (T0) and after (T1) neo-adjuvant chemotherapy. **(A)** regions of interest: frontal (yellow), parietal (red), occipital (orange), temporal (purple) and insular (blue) cortex. **(B)** Robust mixed effects modelling showed relative decreases in frontal glucose metabolism, while increases where observed for parietal for both EC (*n*=100) and FEC (*n*=83) treated patients. Additionally, relative increased temporal and insular metabolism was observed for FEC treated patients and increased occipital metabolism for EC patients. Models were corrected for age, type of scanner and years since chemotherapy. P-values were Benjamini-Hochberg corrected for comparing five outcomes. EC = 4 rounds of epirubicin 90 mg/m^2^ + cyclophosphamide 600 mg/m^2^; FEC = 3-6 rounds of 500 mg/m^2^ 5-fluorouracil and 4 rounds of epirubicin 100 mg/m^2^ + cyclophosphamide 500 mg/m^2^. *pFDR<0.05, **pFDR<0.01, ***pFDR<0.001.

There were no significant differences in injected-activity (*p*=0.201) or time between injection and start scan (*p*=0.360) between the time points. A comparison of relative brain glucose metabolism before and after chemotherapy in the five regions of interest (**Figure 2**, **Table 2** and **Table S1**) revealed time effects for the frontal (ß=-0.350, *p_FDR_* <0.001), parietal (ß=0.273, *p_FDR_* <0.001), and insular cortex (ß=0.180, *p*=0.008). Separately, patients treated with EC (*n*=100) showed decreased frontal (ß=-0.435, *p_FDR_* <0.001), parietal (ß=0.201, *p_FDR_* =0.007), and occipital cortical metabolism (ß=0.348, *p_FDR_* =0.022). In FEC-treated patients (*n*=83), increased relative metabolism was observed in the parietal (ß=0.201, *p_FDR_* =0.001), temporal (ß=0.244, *p_FDR_* =0.048), and insular cortices (ß=0.423, *p_FDR_* <0.001) as well as a decrease in the frontal cortex (ß=-0.220, *p_FDR_* =0.013).

**Table 2:** Robust mixed-effects modelling of time and treatment effects on relative brain glucose metabolism.

	Parameter	ß	(95% CI)	*p*FDR
**Frontal** **cortex**	Time	-0.350	(-0.451, -0.249)	***1.1E-09***
	Chemo regime	0.271	(-0.185, 0.727)	0.306
	Time by Chemo regime	0.215	(0.008, 0.423)	0.110
	EC only: Time	-0.435	(-0.568, -0.302)	***8.2E-09***
	FEC only: Time	-0.220	(-0.38, -0.059)	***0.013***
**Parietal** **cortex**	Time	0.273	(0.166, 0.38)	***3.6E-06***
	Chemo regime	-0.122	(-0.544, 0.299)	0.569
	Time by Chemo regime	0.045	(-0.172, 0.261)	0.686
	EC only: Time	0.256	(0.117, 0.395)	***0.001***
	FEC only: Time	0.301	(0.134, 0.468)	***0.001***
**Temporal** **cortex**	Time	0.076	(-0.074, 0.226)	0.320
	Chemo regime	0.309	(-0.172, 0.79)	0.306
	Time by Chemo regime	0.276	(-0.023, 0.575)	0.121
	EC only: Time	-0.031	(-0.224, 0.161)	0.751
	FEC only: Time	0.244	(0.015, 0.474)	***0.048***
**Occipital** **cortex**	Time	0.213	(0.007, 0.419)	0.056
	Chemo regime	-0.333	(-0.736, 0.071)	0.284
	Time by Chemo regime	-0.321	(-0.736, 0.093)	0.164
	EC only: Time	0.348	(0.076, 0.62)	***0.022***
	FEC only: Time	0.027	(-0.287, 0.341)	0.865
**Insular** **cortex**	Time	0.180	(0.059, 0.3)	***0.007***
	Chemo regime	0.381	(-0.089, 0.851)	0.284
	Time by Chemo regime	0.387	(0.151, 0.622)	***0.008***
	EC only: Time	0.036	(-0.115, 0.187)	0.751
	FEC only: Time	0.423	(0.241, 0.604)	***4.9E-05***

n = 324 scans of 183 subjects. T0 and EC treatments were set as reference levels; therefore, chemotherapy regime effects represent FEC treatment versus EC, and time effects represent changes from T0 to T1. Models were corrected for age, type of scanner and years since chemotherapy (see **Supplementary Materials**). Standardized ß values are presented so that estimates can be interpreted as effect sizes and CI – confidence interval for ß. Benjamini-Hochberg-corrected p-values are presented for comparing the five regions of interest. Bold values means significant effect at the 5% level.

**Figure 3:**
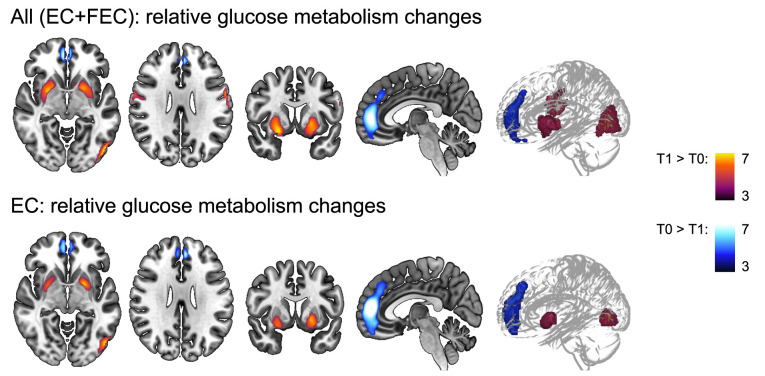
Whole-brain voxel-based analysis of relative glucose metabolism before (T0) and after (T1) neo-adjuvant chemotherapy. General linear modelling showed relative decreased (blue colored T-scale, paracingulate gyrus) and increased (red colored T-scale) frontal (central gyrus) glucose metabolism, as well as increased subcortical (putamen and occipital (lateral cortex) metabolism for EC and FEC treated patients combined. Except for the pericentral gyri decrease, the same regions showed changes in relative metabolism for EC patients separately, but not FEC patients. Models were corrected for age and type of scanner (*n*=282) scans. Statistical significance was inferred with a height threshold of p_FWE_<.05 and a cluster-level p_FWE_<.001. EC = 4 rounds of epirubicin 90 mg/m^2^ + cyclophosphamide 600 mg/m^2^; FEC = 3-6 rounds of 500 mg/m^2^ 5-fluorouracil and 4 rounds of epirubicin 100 mg/m^2^ + cyclophosphamide 500 mg/m^2^.

Two scans were available for 141 subjects and a subsequent whole-brain voxel-based analysis was performed (**Figure 3** and **Table 3**). A relatively decreased metabolism was observed in a cluster covering the majority of the paracingulate gyrus, spreading to the frontal medial cortex (T_peak_=8.03, *p_cluster-FWE_* <0.001, k_E_=1102). Additionally, increased metabolism was observed in the bilateral putamen (T_peak_≥660, *p_cluster-FWE_<*0.001, and k_E_≥613), bilateral central sulcus (T_peak_≥6.18, *p_cluster-FWE_<*0.001, and k_E_≥225), and the left lateral occipital cortex (T_peak_=8.97, *p_cluster-FWE_<*0.001, and k_E_=525). Separately, only patients treated with EC showed a decrease in the paracingulate gyrus (4.5 ± 4.7%) and an increase in left (3.3 ± 4.3%) and right putamen (4.0 ± 4.7%) and left lateral occipital cortical metabolism (3.1 ± 3.6%).

**Table 3:** Significant clusters by whole-brain voxel-based analysis.

k_E_	p_clusterFWE-corrected_	p_peak FWE-corrected_	T	Peak coördinates (mm)			Harvard-Oxford (sub)cortical atlas region	Hemi	Contrast	Peak voxel difference
				x	y	z				
1102	<.001	<.001	8.03	4	52	4	*paracingulate gyrus (frontal lobe)	Both	T0 > T1	-3.2 ± 3.9%
684	<.001	<.001	7.20	24	4	-12	*putamen (subcortical)	R	T1 > T0	3.2 ± 4.1%
613	<.001	<.001	6.60	-26	2	-12	*putamen (subcortical)	L	T1 > T0	2.9 ± 4.4%
525	<.001	<.001	8.97	-50	-80	2	*lateral occipital cortex (occipital lobe)	L	T1 > T0	2.9 ± 3.1%
290	<.001	<.001	6.18	62	-2	30	*precentral gyrus (frontal lobe)	R	T1 > T0	3.3 ± 5.0%
225	<.001	<.001	7.04	-62	-4	26	*postcentral gyrus (frontal lobe)	L	T1 > T0	2.7 ± 3.6%
1648	<.001	<.001	8.99	4	50	10	paracingulate gyrus (frontal lobe)	Both	EC: T0 > T1	-4.5 ± 3.8%
330	<.001	<.001	6.14	26	2	-10	putamen (subcortical)	R	EC: T1 > T0	4.0 ± 4.7%
358	<.001	<.001	6.78	-22	4	-4	putamen (subcortical)	L	EC: T1 > T0	3.3 ± 4.3%
320	<.001	<.001	7.42	-50	-78	0	lateral occipital cortex (occipital lobe)	L	EC: T1 > T0	3.1 ± 3.6%

n = 282 scans of 141 subjects. Age and scanner are included in the statistical models as covariates. *Regions for which mean relative glucose metabolism was calculated for regression analysis.

### Subset shows extreme complaints on self-report

**Figure 4:**
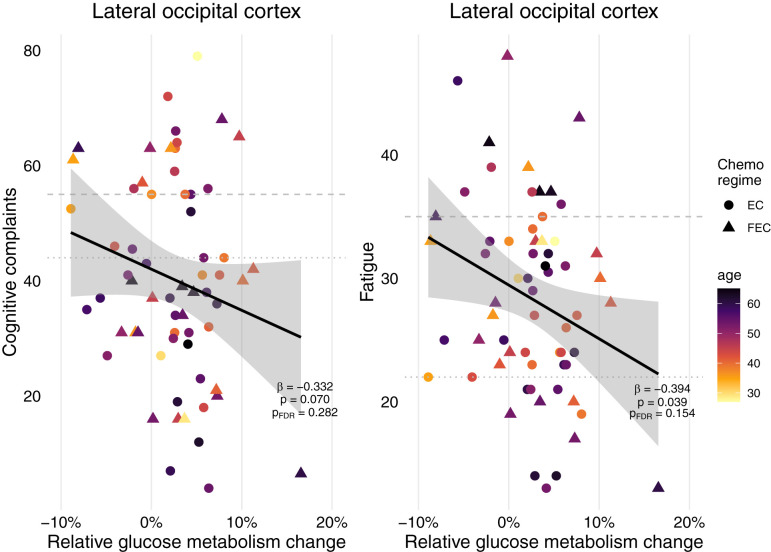
Self-report cognitive complaints and fatigue of patients treated for breast cancer with chemotherapy and their metabolic associations. Relative metabolic changes in the lateral occipital cortex preceded fatigue years later, but not cognitive complaints. After FDR-correction, this association disappeared. No associations were found for changes in the frontal cortex (paracingulate cortex, pericentral gyri) or basal ganglia (putamen), nor for absolute baseline metabolism in any of these regions or having received additional radiotherapy, endocrine therapy and/or HER2 therapy (*n*=64). Final linear models included chemo regime and were corrected for age, and years since treatment. Striped line = extreme cognitive complaints/fatigue, dotted line = cognitive complaints/fatigue. EC = 4 rounds of epirubicin 90 mg/m^2^ + cyclophosphamide 600 mg/m^2^; FEC = 3-6 rounds of 500 mg/m^2^ 5-fluorouracil and 4 rounds of epirubicin 100 mg/m^2^ + cyclophosphamide 500 mg/m^2^.

When assessing various everyday cognitive complaints, 38% of survivors indicated experiencing complaints and 27% indicated severe complaints (**Table 1**). Regarding fatigue, 80% of the patients experienced fatigue and 23% experienced severe fatigue. When evaluating the association of frontal (paracingulate gyrus, pericentral gyri), occipital (lateral cortex) or basal ganglia (putamen) brain metabolic changes throughout chemotherapy with complaints years later, only occipital cortex changes showed a significant association with fatigue, but not cognitive complaints (**Figure 4**). However, this trend disappeared after FDR correction. Neither baseline metabolism, as well as having received endocrine, radiotherapy and/or HER2 therapy influenced fatigue or cognitive complaints (data not shown).

## Discussion

To our knowledge, this is the first *in-vivo* study to investigate longitudinal changes in cerebral metabolism shortly after chemotherapy in breast cancer patients. This study aimed to evaluate changes in brain glucose metabolism during chemotherapy, as well as their predictive value for cognitive and psychological/functional complaints in breast cancer patients, based on FDG-PET/CT imaging. First, we found relatively decreased metabolism in the frontal cortex (paracingulate gyrus), while increased metabolism was observed in other parts of the frontal cortex (central gyrus), parietal, occipital, temporal, and insular cortices, and in the basal ganglia (putamen). Changes in the frontal (paracingulate gyrus), temporal, occipital, insular, and basal ganglia were dependent on chemotherapy regimen. Second, although ¼ of the women experienced extreme cognitive complaints or fatigue seven years after the end of chemotherapy, cerebral metabolic patterns throughout chemotherapy, endocrine therapy, and/or radiotherapy were not associated with these complaints.

### Underlying mechanisms of FDG-PET uptake

It is well established that cerebral FDG uptake is proportional to the brain energy consumption (10). The utilization rate of glucose, the major source of energy in the brain, is associated with neuronal activity and hence provides the basis for interpreting FDG-PET data. However, recent studies have shown that non-neuronal activity can also contribute to FDG signaling, potentially masking the underlying deficits in the brain. *In vivo* evidence exists for widespread astroglial glutamate transport that substantially affects FDG-PET uptake in the rodent brain (30). Additionally, microglial cells rely on an efficient glucose transport system capable of extracting high glucose levels even in regions with an insufficient supply of neurons/astrocytes to survive (13, 14). Therefore, our observed differences in glucose utilization could reflect changes in the activity/presence of either of these cell populations or resulting network changes.

First, inflammatory processes may have been involved. In a recent study by our group, more activated microglial cells were observed *via* TSPO-PET, mainly in the parietal and occipital regions and to a lesser extent in the frontal cortex, when comparing patterns in breast cancer patients shortly after chemotherapy to chemotherapy-naïve patients or healthy women (8). This could indicate that the increased relative parietal, pericentral, and occipital hypermetabolism observed in this study was caused by inflammatory processes (13). Indeed, the patterns of increased metabolism observed here are reminiscent of the hypermetabolism observed in the basal ganglia in invasive fungal-infections (31), suggesting an inflammatory reaction spreading to the putamen. As chemotherapeutic agents are known to induce peripheral inflammation with increasing cytokine levels (32, 33), cytokine upregulation may, in part, be responsible for changes in brain glucose metabolism. Indeed, after interferon-α therapy, increased basal ganglia glucose metabolism was observed, while frontal metabolism was reduced (34). Interferons are innate immune system cytokines that induce behavioral alterations such as fatigue (34). Research suggests that interferon-α can alter functional basal ganglia circuitry (34) *via* dopaminergic activity (35), consequently contributing to reduced frontal metabolism (36), both observed in our cohort. Second, the regional loss of neurons/synaptic density may have influenced the decrease in the relative glucose metabolism. Ample magnetic resonance imaging (MRI) studies have demonstrated (subtle) GM volume losses, mainly in the frontal and temporal regions, after chemotherapy (37–39). Therefore, partial volume effects due to GM atrophy or decreased neuronal density, resulting in the loss of energy demands (40), could explain the decreased glucose utilization observed in the frontal cortex. We were unable to investigate this hypothesis in this cohort, because no MR data were available. Thirdly, regional loss of neurons can induce compensatory distant hyperactivity. In patients with Parkinson’s, neurodegeneration of dopaminergic neurocircuits is believed to underlie the hyperactivity observed in the basal ganglia (34). This disinhibition leads to bursts of neuronal activity and metabolic hyperactivity. A change in efficient network communication. e.g., diaschisis due to (non-)focal pathophysiological events, may therefore also underlie the observed metabolic changes (41, 42). This inefficient network organization is further underscored by MRI findings, showing widespread structural differences in white matter after chemotherapy, potentially impairing the ability of the white matter to rely information (38). Why regional changes occurred, could be potentially explained by neural and oligodendrocyte precursor cells being particularly sensitive to toxicity of chemotherapy (43, 44). With adult neurogenesis occurring primarily in niche regions (e.g., basal ganglia) (45), migration of precursor cells to injured regions could underscore the time-dependency of compensatory mechanisms in locations farther from or less connected to these niche regions. Previous studies have indeed underscored widespread brain changes after treatment for cancer (6, 37–39). Lastly, in longitudinal follow-up, the effect of aging should also be considered. Metabolic activity is especially affected by aging in the frontal lobes (46), which was also observed in our cohort and was restricted to the frontal cortex.

In conclusion, changes in glucose metabolism observed in this population could reflect changes in the number of active/available neuronal or inflammatory cells, a shift in circuitry, or a combination of both. However, we were unable to distinguish between these processes. In combination with our earlier findings of glial hyperactivation in the parietal/occipital cortex using TSPO-PET (8), the results presented here underscore the inflammatory pathways in these regions, potentially spreading to the frontal/temporal/insular cortex and the basal ganglia.

### Systemic chemotherapy induces differential metabolic patterns

Previous cross-sectional studies in chemotherapy-treated breast cancer patients have found no (17, 18) or subtle relative (19) differences in glucose metabolism, with widespread (though mainly frontal) glucose hypometabolism when compared with screened healthy individuals. The results presented here partly confirm and add to these findings: decreased frontal glucose metabolism and increased parietal, temporal, insular cortical, and basal ganglia metabolisms. Furthermore, we observed that treatment with EC or FEC did not induce contradictory effects but presented complementary results, suggesting that similar (compensatory) mechanisms may be at play. Both treatment regimens consisted of a combination of anthracycline (epirubicin = E), an alkylating agent (cyclophosphamide = C) and three–12 rounds of a plant alkaloid (paclitaxel). The only difference between the two regimens was the addition of an anti-metabolite (5-fluorouracil [F]) to FEC. Preclinical *in vivo* studies have provided valuable evidence for disentangling neurotoxicity induced by single agents. Fluorouracil can cross the blood-brain barrier and induce direct CNS damage (apoptosis) as well as delayed damage to white matter tracts, while only showing acute/sporadic inflammation, additionally involved in neurogenesis/gliogenesis (47, 48). Cyclophosphamide can induce oxidative stress and is involved in neurogenesis/gliogenesis (48). Although both E and C are unable to cross the BBB at high concentrations (7), they can increase BBB permeability, enabling direct interactions (49). Moreover, at sublethal doses to cancer cells, E induces microglial activation (50), suggesting that treatment with EC is sufficient to induce an inflammatory reaction [observed here and earlier in the parietal/occipital cortex (8)]. The addition of fluorouracil to the chemo-cocktail induced additional brain responses, as reflected by temporal and insular, but not occipital, relative hypermetabolism, suggesting that inflammatory (E) and oxidative stress (E + C) responses alone are insufficient to change metabolism in these regions, but rather require changes in cell proliferation (F + C) (48). The first study investigating long-term effects in breast cancer patients found that chemotherapy alone (unspecified, however) was not associated with decreased metabolism, whereas this was the case for tamoxifen+chemotherapy, relative to chemotherapy-alone or untreated subjects five–ten years after chemotherapy (basal ganglia) (18). As endocrine or external radiation therapy was administered after FDG-PET/CT imaging, these complementary therapies did not influence the changes observed. Additionally, a recent prospective study in patients with lung cancer treated with platinum-based chemotherapy observed decreases in absolute glucose metabolism (~20%) (20) across all investigated GM structures, including the parietal/cerebellar cortices and basal ganglia, with peak reduction in the frontal cortex. This widespread GM reduction aids in the observation of relatively increased metabolism. In concordance, we observed the largest differences in the relative frontal reduced resting metabolism (approximately 5%). Moreover, reduced metabolism in the frontal cortex could be interpreted as the driving mechanism of the observed changes; widespread relative increases could reflect normal physiology, while other regions (e.g., the frontal cortex) show significant hypometabolism, resulting in *relative increases* in other brain regions. Taken together, these findings demonstrate the chemotherapy-specific effects on brain metabolism. Moreover, because clinical FDG PET/CT is scheduled for staging in high-risk patients (51) (higher probability of more aggressive tumors, metastases, or recurrence with poor overall survival), the disease stage could also have influenced the observed metabolic changes. Further dedicated neuroimaging research is necessary to disentangle the potential neurotoxicity of specific agents or to disentangle disease from treatment effects.

### Subset shows extreme complaints on self-report

In concordance with earlier studies (3, 4), ¼ of the women reported extreme cognitive complaints or fatigue, on average, seven years post-chemotherapy. The above-mentioned mechanisms could underlie such complaints; however, this was not observed in our analysis. In contrast, an earlier study showed that short-term memory complaints are associated with cortical metabolism in cancer patients (17), not excluding metabolic changes as an acute compensatory mechanism. Moreover, several psychological measures have been shown to be related to cognitive complaints such as depression and anxiety (52, 53), which could be interesting for further evaluation in the context of cerebral metabolism. Additionally, while fatigue is one of the most reported side effects of treatment for non-CNS cancer, associations between brain metabolism and fatigue have not yet been examined. However, the increased basal ganglia metabolism observed after interferon-*α* therapy was previously shown to be associated with fatigue (energy subscale) assessed immediately after therapy (34). Combined, this could indicate that acute changes measured in glucose metabolism could be predictive of short-term complaints, but not of long-term complaints, which are potentially driven by other delayed processes, such as the decreased ability of myelin to relay information (47, 54). Alternatively, more dynamic measurements of behavioral outcomes could unravel more subtle changes associating with short-term metabolic differences, since we did observe a trend for short-term occipital metabolism changes preceding fatigue complaints years later.

### Limitations and future research

Although the use of baseline/follow-up clinical PET/CT provides a valuable dataset for assessing metabolic changes, this study had some limitations. First, the patients were scanned using three different scanners (resulting in different spatial resolutions), which could influence quantification, although the scanner was used as a covariate. Moreover, because spatial resolution differences are small [6-7 mm (older systems) to 4-5 mm (newest systems]) relative to the size of the ROIs or smoothing kernel for voxel-based analyses, these effects are expected to be second-order. Second, normalizing uptake to mean cortical GM uptake ensured that global uptake differences could be partly considered in the statistical models. However, when normalizing values to a (pseudo-reference) region, we were unable to identify whether regional hypo/hypermetabolism truly reflects decreased/increased metabolism or is driven by absolute metabolic changes in the surrounding GM regions. Although methods exist to approach absolute quantification (55) (e.g., determining plasma kinetics with an arterial-plasma input function), they are invasive and unnecessary in clinical examinations. Third, since no MR data were available, we were unable to perform partial volume correction, which may have influenced our results, especially by chemotherapy-induced volumetric effects or aging in longitudinal follow-up (40, 56). Fourth, no comparative healthy control data were available for the large cohort. Such data would allow group characterization of baseline differences and a more accurate evaluation of the observed intensity differences compared to test-retest variability in a healthy population. Lastly, a single time point questionnaire measurement was conducted, to which only ½ patients responded, which could have introduced reporting bias. Nonetheless, our results still demonstrated that a minimum of 1/8 women reported extreme cognitive and/or fatigue complaints. Future studies including more dynamic measurements of psychological complaints would be valuable in determining associations, potentially capturing more subtle complications in patients.

In conclusion, this hypothesis-generating study provides evidence of frontal hypometabolism and potential inflammatory mechanisms in the parietal and occipital cortices of patients treated for breast cancer with chemotherapy. While early metabolic changes were not associated with long-term complaints, we showed that clinical FDG-PET can provide valuable and low-threshold initial biomarker investigation, potentially linked with other or more short-term complaints. Additionally, this study requires further follow-up studies using dedicated PET(-MR) neuroimaging to better characterize metabolic and neuroinflammatory changes, preferably combined with elaborate baseline patient characterization.

## Data availability statement

The original contributions presented in the study are included in the article/**Supplementary Material**. Further inquiries can be directed to the corresponding author.

## Ethics statement

The studies involving human participants were reviewed and approved by the Ethical Committee Research UZ/KU Leuven. The patients provided their written informed consent to participate in the prospective part of this study.

## Author contributions

GwS: Conceptualization, Methodology, Software, Validation, Formal Analysis, Investigation, Data Curation, Writing -original Draft, Writing – Review & Editing, Visualization, Project administration, Funding acquisition; GeS: Methodology, Software, Validation, Formal analysis, Data Curation, Writing – Review & Editing; DVW: Conceptualization, Validation, Investigation, Writing – Review & Editing; NL: Methodology, Software, Validation, Writing – Review & Editing, Visualization; TVC: Software, Writing – Review & Editing; JB: Conceptualization, Software, Writing – Review & Editing; MK: Validation, Writing – Review & Editing; AS: Conceptualization, Resources, Writing – Review & Editing, Supervision, Project administration; KL: Conceptualization, Resources, Writing – Review & Editing, Supervision; SS: Conceptualization, Software, Validation, Resources, Writing – Review & Editing, Supervision, Funding acquisition; SD: Conceptualization, Methodology, Validation, Resources, Writing -original Draft, Writing – Review & Editing, Supervision, Project administration, Funding acquisition. All authors contributed to the article and approved the submitted version.

## Funding

GwS is supported by Research Fund KU Leuven (C24/18/067) and Stichting Tegen Kanker, JB is supported by Research Foundation Flanders (FWO 11B9919N). NL is supported by Research Fund KU Leuven (C14/18/096). TVC is supported by Research Fund KU Leuven (C24/18/095). Funding sources provided a financial contribution.

## Conflict of interest

The authors declare that the research was conducted in the absence of any commercial or financial relationships that could be construed as a potential conflict of interest.

## Publisher’s note

All claims expressed in this article are solely those of the authors and do not necessarily represent those of their affiliated organizations, or those of the publisher, the editors and the reviewers. Any product that may be evaluated in this article, or claim that may be made by its manufacturer, is not guaranteed or endorsed by the publisher.
