# Supplementary material for: Cerebral glucose changes after chemotherapy and their relation to long-term cognitive complaints and fatigue
Source: Front Oncol. 2022 Oct 13;12:1021615. doi: 10.3389/fonc.2022.1021615 (PMC9612406; doi:10.3389/fonc.2022.1021615)
Supplement: Supplementary file 1 [file Table_1.docx]

# Supplementary materials

### Table S1. Mixed-effect modeling of time and treatment effects on relative brain glucose metabolism, intercept, and covariate outcomes.

|  | **Parameter** | ß | (95% CI) | *p* |
| --- | --- | --- | --- | --- |
| **Frontal**  **cortex** | Intercept | 0.069 | (-0.164, 0.302) | ***7.0E-01*** |
|  | scanner MI Discovery | -0.342 | (-0.632, -0.051) | ***0.027*** |
|  | scanner Biograph 16 | 0.205 | (-0.045, 0.456) | 0.181 |
|  | age | -0.369 | (-0.486, -0.252) | ***2.1E-08*** |
|  | years since treatment | -0.053 | (-0.28, 0.175) | 0.650 |
| **Parietal**  **cortex** | Intercept | -0.116 | (-0.333, 0.102) | ***5.0E-01*** |
|  | scanner MI Discovery | -0.585 | (-0.885, -0.286) | ***0.001*** |
|  | scanner Biograph 16 | -0.029 | (-0.283, 0.225) | 0.824 |
|  | age | -0.111 | (-0.219, -0.003) | 0.075 |
|  | years since treatment | 0.110 | (-0.101, 0.322) | 0.501 |
| **Temporal**  **cortex** | Intercept | -0.211 | (-0.464, 0.042) | 0.260 |
|  | scanner MI Discovery | 0.195 | (-0.203, 0.594) | 0.337 |
|  | scanner Biograph 16 | 0.184 | (-0.146, 0.514) | 0.343 |
|  | age | -0.133 | (-0.256, -0.01) | 0.075 |
|  | years since treatment | -0.132 | (-0.377, 0.113) | 0.501 |
| **Occipital**  **cortex** | Intercept | -0.008 | (-0.239, 0.222) | 0.944 |
|  | scanner MI Discovery | 0.556 | (0.117, 0.996) | ***0.023*** |
|  | scanner Biograph 16 | -0.456 | (-0.799, -0.112) | ***0.024*** |
|  | age | 0.077 | (-0.025, 0.18) | 0.143 |
|  | years since treatment | -0.093 | (-0.309, 0.123) | 0.501 |
| **Insular**  **cortex** | Intercept | -0.304 | (-0.546, -0.061) | 0.075 |
|  | scanner MI Discovery | -0.438 | (-0.775, -0.101) | ***0.023*** |
|  | scanner Biograph 16 | 0.621 | (0.336, 0.907) | ***1.3E-04*** |
|  | age | 0.098 | (-0.022, 0.219) | 0.139 |
|  | years since treatment | -0.340 | (-0.576, -0.103) | ***0.026*** |

Note. n = 324. T0 and scanner Biograph 40 are set as reference levels. Standardized ß’s are presented so estimates can be interpreted as effect sizes, CI – confidence interval for ß. Benjamini-Hochberg corrected p-values are presented, for comparing five regions of interest.
